# Indicators of newborn screening for congenital hypothyroidism in Sri Lanka: program challenges and way forward

**DOI:** 10.1186/1472-6963-14-385

**Published:** 2014-09-12

**Authors:** Manjula Hettiarachchi, Sujeewa Amarasena

**Affiliations:** Nuclear Medicine Unit, Faculty of Medicine, University of Ruhuna, Galle, Sri Lanka; Department of Pediatrics, Faculty of Medicine, University of Ruhuna, Galle, Sri Lanka

**Keywords:** Newborn screening, Congenital hypothyroidism, Sri Lanka, Program challenges

## Abstract

**Background:**

Many of the countries in the Asia Pacific Region are just initiating newborn screening programs for selected metabolic and other congenital disorders. The present study is aimed at evaluating the congenital hypothyroidism screening program in the Southern region of Sri Lanka in terms of coverage, effectiveness of detecting and managing the cases.

**Methods:**

The Newborn Screening System Database of Sri Lanka was reviewed from January 2011 to December 2012. The data of 47 babies who tested positive for hypothyroidism were analyzed.

**Results:**

Total of 78,167 babies (99.0% of live births) were screened. Of them, 5.8% (n = 4,472) were discharged within 12 hrs of delivery where as 58.1% (n = 44969) were discharged afterwards but within next 12 hrs (i.e., day 1). The positive predictive value for congenital hypothyroidism (CH) was 9.0%. The incidences of primary CH among screened infants were 1:1682. False positive rate among screened infants was maintained below 0.70%. Mean age of serum confirmation was 23.8 (±8) days.

**Conclusions:**

In the light of the present findings, we would suggest direct communication systems, linking newborn screening program to the family unit. This would enhance timely follow-up for screen-positive infants and facilitate information sharing. Establishing a program with, public-private sector partnership should be considered. Costs could be contained if the specimen collection, its transportation and communication are carried out by this partnership and the laboratory tests are conducted by a non-profit organization such as a University in order to achieve the goal of universal coverage.

**Electronic supplementary material:**

The online version of this article (doi:10.1186/1472-6963-14-385) contains supplementary material, which is available to authorized users.

## Background

Congenital hypothyroidism (CH) is the most common preventable cause of mental retardation [1]. Screening programs enable this potentially devastating disease to be detected at a very early stage and allows treatment to be started promptly, before any significant damage is caused [2]. CH screening in newborns and early levothyroxine (L-T4) substitution has dramatically improved the outcomes of intellectual potential and linear growth in children with CH [3–5]. Within the first few days of life, drops of blood are collected from a newborn’s heel on to a filter paper and are analyzed. Early detection and channeling resources to provide appropriate services to the most deserving infants is the objective of the screening program. Every effort has been made to ensure that every newborn receives quality appropriate services within the scope of the screening program [6].

Many of the countries in the Asia Pacific Region are just initiating newborn screening programs for selected metabolic and other congenital disorders [7]. Newborn dried blood spot screening (NDBS) as a public health improvement strategy has existed in some countries in the Asia Pacific since the 1960s (Australia, Japan, New Zealand). Despite attempts over time to begin organized newborn screening in various countries in the region, its implementation has been slow, mainly due to economic factors. The Ministry of Health of Sri Lanka has endorsed the introduction of screening program for CH using the TSH assay methods (Radioimmunoassay –RIA or Enzyme Linked Immunosorbant Assay –ELISA) in the Southern region of the country. Program evaluation is an important organizational practice in the public health sector. However, it is neither practiced consistently, nor is it sufficiently well-integrated into the day to day management of most programs. Therefore, the present study is aimed at evaluating the congenital hypothyroidism screening program in the South of Sri Lanka in terms of coverage, effectiveness of detection and managing the cases.

## Methods

The study was carried out through follow-up and retrospective approaches at the Nuclear Medicine Unit. The study protocol received approval from the Institutional Ethical Review Board of the Faculty of Medicine, University of Ruhuna. The Newborn Screening program of the Southern Province began in September 2010 with the circular issued by the Director General of Health Services of Sri Lanka and the circular is attached as an Additional file 1.

The procedure followed in the hospitals is that once a newborn assessment has been conducted mother-infant pair is discharged from the hospital. Therefore, blood spot collection should be carried out prior to the hospital discharge. The cut off values for blood spot TSH used was 40.0 mIU/L upto 48 hrs (i.e., Day 2) and 20.0 mIU/L after 48 hrs of life (i.e., Day 3 onwards). These figures were based on the analysis of pilot study [8] in the region. The blood spot analyses were done using the IMMUCHEM™ NEONATAL TSH-MW ELISA (bulk kit −20 plates) and RIA kits (500 tubes kit) provided by MP Biochemicals, USA. Once a positive sample is found a repeat test was also performed to confirm results. Then the parents were contacted immediately in order to get a serum sample for the confirmation of the disease. Both serum TSH and free thyroxine levels were determined in these babies using respective ELISA kits provided by the MP Biochemicals, USA. Congenital hypothyroidism was confirmed if serum TSH >9.8 mIU/L and free thyroxine < 10 pmol/L. Then the subjects were referred for treatment and follow up.

The Newborn Screening System Database of the NMU (nsisd.ruh.ac.lk) was reviewed from January 2011 to December 2012 to calculate the number of live births and screened infants. In addition, the number of total positives (true positives and false positives) was recorded to calculate the positive predictive value of the screening test. These data were used to estimate the coverage of the program. Further, screening results including age at screening, confirmation, and age at starting treatment were also reviewed.

## Results

The public healthcare system in the Southern Province of Sri Lanka is conducted mainly, with the services of a Teaching Hospital, two General Hospitals, eight Base Hospitals where board certified pediatricians are available for neonatal care. Further, there are 60 Divisional Hospitals for maternal care with delivery facilities. Even though the screening program began in September 2010, the sample collection was not well established in all hospitals. It took 2–3 months to streamline the process of sample collection, transporting to the testing facility at the Nuclear Medicine Unit and delivering the results. Therefore, the data for the year of 2010 was not included in this study (4680 babies were screened and 4 babies were confirmed as having primary CH out of 98 positive cases) during this period. Further, another General Hospital adjacent to Southern Province (Monaragala) was included into the program from February, 2011 onwards but it was not included in the analysis (10,592 babies were screened and 8 true positives during these 2 years.

The program indicators for Southern Province are summarized in Table 1. We were able to achieve 99% coverage among live births within a year of implementation and maintained it throughout the study period. By the end of 2012, 47 true positive infants were identified with a positive predictive value of 9.0%. The rate of incidences of primary CH among screened infants was 1:1682. False positive rate among screened infants were maintained below 0.70%.

**Table 1:** **Program indicators**

	2011	2012	Total
Total number of live births	38419	39285	77704
Screened infants	38248	39113	77361
Percentage of coverage	99.6%	99.6%	99.6%
Total positives	303	235	538
True positives	21	26	47
False positives	283	209	492
Positive predictive value	6.9%	11.1%	8.6%
Incidence of primary CH among screened	1:1821	1:1504	1:1682
False positive rate among screened	0.74%	0.53%	0.64%

Further, analyses done on sample collection and data are summarized in Table 2. Our records indicated that 5.8% (n = 4,472) of babies’ blood spots were collected within 12 hrs of delivery, and 58.1% (n = 44969) were collected in the following 12 hr period (i.e., within day 1; Table 2). The number of blood spots collected on day 2 was 18.8% (n = 14516) and day 3 was 8.2% (n = 6315) respectively. Only 9.2% (n = 7090) of babies’ blood spots were collected after 72 hrs of life (i.e., from Day 4 onwards).

**Table 2:** **Blood spot TSH level (mIU/L) distribution according to the date of blood spot collection**

		Sample collected on				
		< 12 hrs	Day 1	Day 2	Day 3	After Day 4
		(n = 4472)	(n = 44969)	(n = 14516)	(n = 6315)	(n = 7090)
Normal infants	n	4359	44640	14487	6300	7037
	Median	7.3	6.2	2.8	1.8	2.1
(n = 76823)	Range	1.0 – 39.9	1.00 – 39.9	1.00 – 39.7	1.00 – 18.2	1.00 – 19.3
False positives	n	112	301	23	15	41
	Median	47.3	47.2	61.9	53.4	53.4
(n = 492)	Range	40.0 – 268.7	40.0 – 319.4	40.2 – 312.5	24.7 – 121.5	20.1 – 258.4
True positives	n	1	28	6	--	12
	Median	230.10	191.4	204.7	--	189.3
(n = 47)	Range		48.4 – 342.1	81.0 – 370.9	--	32.4 – 409.6

The sample collection ages ranged from 12 hours of age to 83 days of age. The median age of screening sampling in the program was 24 hours of age (i.e., day 1). In this program, the age at serum sampling (for the confirmatory testing) was conducted between 9.0 to 45.0 days with a mean of 23.0 ± 8.0 days. The serum confirmation was made before 21 days of age for 51.1% (n = 24) of the cases. The confirmation was made between 22 days and 28 days of age for 21.3% of the cases and the confirmation was made after 4^th^ week of age for 27.6% (n = 13) of the cases. Thyroxine replacement treatment commenced when the serum confirmed results were available. The mean age for the start of the treatment was 25.4 ± 8 days.

## Discussion

Sri Lanka is the only Asian country which provides free government funded healthcare to all its citizens. This program came into existence in 1948 when the country gained independence from the British Empire. Probably as a consequence, the country has the lowest mortality rate in the South Asian region. The country as a whole has about 350,000 live births per year. About 11.0% of these births are in the Southern Province of the country. The universal coverage of the healthcare system in Sri Lanka, results in all births (99.9%) taking place in a hospital or in a maternity home. These facilities are staffed with trained medical practitioners and public health midwives (PHM). There were no reported home deliveries during the study period in Southern Province.

In the developing countries, newborn screening programs are yet to achieve full nationwide coverage and the programs in existence are generally mediocre due to inherent weaknesses in the social service infrastructure [9]. Nevertheless, this regional program initiated in 2010 had achieved almost 99.0% coverage within 4 months of its implementation. This is a noteworthy accomplishment for a developing country. It points to the feasibility of conducting such a program in any other developing country. In developed countries, the program coverage exceeds 99.5% [10].

The magnitude of false-positive results generated in newborn screening programs, particularly for congenital endocrinopathies, presents a great challenge for future improvement of this important public health program [11]. In this program there was a marked decrease in the number of total positive cases (303 in 2011 to 235 in 2012) and false positive rate (0.74% to 0.53% in 2011 and 2012 respectively) among screened infants. The number of true positive cases and consequently the incidence of CH were increased from1:1800 in 2011 to 1:1500 in 2012. The improved performance of the test could be attributed to experience gained in diagnosis and treatments, and improved analytical methods such as radioimmunoassay and enzyme linked immunosorbant assay. On average we were able to identify 1 true positive result out of 14 false-positives in 2011; and it improved to 1 positive result out of 8 false-positives in 2012. Higher number of false positives resulted in higher number of tests that had to be repeated, giving rise to higher costs for the program. Ability to obtain repeated (recall) blood specimens was unpredictable, and generally success was much less than 100%. The negative psychological effect of these false-positive results on parents and families is worthy of attention [12].

The appropriate age of sampling in the newborn screening program was a matter of debate. The optimum age of sampling depends on many factors like the number of diseases screened for and screening method [9]. The American Academy of Pediatrics recommended that every infant should be tested before discharge from the nursery, optimally by 48 hours to 4 days of age. However, screening before hospital discharge or before blood transfusion was preferable to avoid missing the diagnosis of hypothyroidism. False-negative results may occur by screening a very sick newborn or after blood transfusion [13]. But, in most of developing countries including Sri Lanka, a significant problem is the early discharge of newborns from maternity hospitals, typically before 24 hours [14]. It has been speculated that the specimens collected in the first 24 to 48 hours of life resulted in higher false-positive rates [15]. We were able to minimize false-positives by using a higher cut-off value. We introduced a new cut off value for samples collected prior to 72 hours (i.e., Day 3) as 40 mIU/L based on the results of a pilot study [8]. Our decision was justified as the lowest TSH value for a true positive baby whose blood spot collected on Day 1 was 48.4 mIU/L (Table 2). If this new value of 48.0 mIU/L is applied to the data base, 230 babies (blood spot TSH 40.0 to 48.0 mIU/L) would not be subjected to recalling, and the number of false positives of the program would be 262 subjects. Further, the false positive rate would be 0.34%.

The possibility of encountering false negatives with the higher cut-off value of 40 mIU/L needs to be examined. Unfortunately there was no follow-up study to verify the likelihood of this occurring. Some programs have a single TSH cutoff, while other programs have age-related cutoffs. For example, specimens obtained in the first 24 h of life may have a TSH cutoff of >60 mU/L, whereas specimens obtained after 72 h have a TSH cutoff of >15 mU/L [16]. Lott et al., [17] developed age-related reference (cutoff) values and an algorithm to identify babies at risk of CH using Auto DELFIA analysis based on the manufacturer’s recommended cutoffs for TSH. A value of ≥34.0 mIU//L was considered to be abnormal for babies who were 0 to 47 hrs old and the figure of ≥28.0 mIU/ L was considered as abnormal for babies ≥ 48 hrs old. They concluded that high TSH in babies <24 hrs old was unreliable for screening newborns for hypothyroidism and that the infant should be at least 48 hrs old for TSH and T_4_ testing. If not, the cutoff value must be set to a higher value to prevent getting excessive number of false-positive results; however, this increases the chance of missing a truly hypothyroid baby. Further, Mengreli et al., [18] reported that using a TSH cutoff point of 10.0 mIU/L whole blood, 56 additional infants with CH were diagnosed when blood samples collected on fifth day of life were analyzed using in house RIA TSH method. It is known that changes in threshold limits influence the number of false-positive and false-negative results [19]. The lowering of the cutoff point by 10 mIU/liter has increased 10 times the number of children requiring re-evaluation [18] in whom the diagnosis of hypothyroidism was not confirmed (false positive results).

Re-testing raises organizational challenges to the healthcare system and also it takes an emotional toll on the parents. Lowering TSH cutoffs will result in increased costs to NBS programs (primarily through higher recall rates), yet it is not clear that the additional, milder CH cases benefit from early detection and treatment [20]. A study from Sweden found that cases of “subclinical CH” had an average IQ decrement of 7 points [21]. Each one-point drop in IQ is estimated to effect a 1% reduction in lifetime earnings [22].

The incidence of the severe forms of thyroid dysgenesis, aplasia, hypoplasia/ectopia, has remained relatively constant (1:4259), despite the lowering of the TSH cutoff [23]. On the other hand, several studies report that detection of milder forms of hypothyroidism, in particular “thyroid-in-situ”, and, to a lesser extent, dyshormonogenesis and ectopic thyroid glands, account for the majority of additional cases leading to the increased incidence of CH [24]. In the Quebec, Lombardy, and New Zealand NBS programs, the incidence of more severe forms of thyroid dysgenesis were unchanged before and after the lowering of the TSH cutoff values [25].

As NBS programs gained experience with detection of neonates with CH, some elected to lower the screening TSH cutoff levels. Lowering of the TSH cutoff led to a higher incidence of CH, primarily explained by the detection of milder cases, many characterized by a eutopic, normally formed thyroid “gland-in-situ” [24]. Detection of milder forms of CH has refocused attention on the initial intent of NBS, which is prevention of mental retardation. Lowering of the TSH cutoff increased the labor and economic burden of NBS programs. Moreover, it is not clear that these milder (often transient) cases of CH benefit from early detection and treatment [26]. The additional cost from the increased number of retesting amounts to about 1.8% of the screening budget [18]. Therefore, we wish to reassess the cut-off values used in this program and more importantly enhance the analytical technique using time–tested fluoroimmunoassay in place of RIA/ELISA.

The median age of screening sampling in the program (1.0 day) was one of the deviations from programs of other developing countries (mean age of 4.7 to 5.3 days) [8, 15]. In this program, the age at serum sampling was conducted between 9.0 to 45.0 days with a mean of 23.0 ± 8.0 days. However, other countries had much lower period i.e., at 9.4 days (range 7–21 days) in Alexandria [9], 15.4 days (range 6–23 days) in UAE [27]. The delay that we experienced was due to issues pertaining to the recalling system in both rural and urban settings. The delay in serum sampling in this program is due to the logistics of getting the samples to the testing facility. Further, delay in diagnosis and treatment was seen among the group of infants whose blood spot collection has been postponed due to other factors such as collecting sample after Day 4.

The implementation of this program was met with considerable challenges due to paucity of resources. Generally, essential supporting services and supplies were lamentably inadequate. Frequently laboratory services were interrupted due to lack of reagents. A critical issue is the lack of adequate trained personnel. In many an instance a healthcare facility is manned by a single qualified person. Hence the program is often held up if that person happens to be to on leave. In spite of these obstacles, recognition of the immense benefit that flows from a program such as this should inspire us to seek solutions for assisting the most vulnerable of the citizenry.

## Conclusions

Efficient data collection and communication between parents and the medical service providers can be improved with help of modern technologies. To overcome the inefficiencies of the rural postal service and the poor transportation system, a web based tool is being considered for data collection; internet short message system (SMS) is proposed for communication between the parents and medical personnel. These measures would help link the screening program directly to the family unit and bring about timely follow-up of infants who prove positive for CH and help provide remedial measures quickly. To accomplish these endeavors, the services of internet service providers and the mobile phone service providers should be enlisted into the screening program. These and other improvements for the screening program as well as working towards the goal of achieving nationwide coverage require devoting considerable financial resources and time. To advance the plan of action, establishing a program with a public-private sector partnership should be considered. Costs could be curtailed if the specimen collection, its transportation and communication are carried out by this partnership and the laboratory tests are conducted by a non-profit organization such as a University. The participants will have to pay a modest fee for this service, with subsidies for the needy. With this approach, while tapping into efficiencies of the private enterprise, the goal of universal coverage could be achieved speedily with reduced costs.

## Consent

Written informed consent was obtained from the patient for the publication of this report and any accompanying images.

## Electronic supplementary material

Additional file 1: **Circular NBS.** http://www.biomedcentral.com/imedia/1284229052108242/supp1.pdf. (PDF 597 KB)
