# Supplementary material for: Indicators of newborn screening for congenital hypothyroidism in Sri Lanka: program challenges and way forward
Source: BMC Health Serv Res. 2014 Sep 12;14:385. doi: 10.1186/1472-6963-14-385 (PMC4167276; doi:10.1186/1472-6963-14-385)
Supplement: Supplementary file 1 — Additional file 1: Circular NBS. http://www.biomedcentral.com/imedia/1284229052108242/supp1.pdf. (PDF 597 KB) [file 12913_2013_3478_MOESM1_ESM.pdf]

13.8.2010

Ministry of Health
Baddegama Wimalawansa Thero Mawatha
Colombo.

Circular Letter No; 02 - 30/2010

All DDGs Ministry of Health
Provincial Director of Health Services - Southern Province
All Directors of Teaching Hospitals - Southern Province
Regional Directors of Health Services - Southern Province
All Heads of Institutions - Southern Province
All Medical Officers of Health - Southern Province

Newborn Screening for Congenital Hypothyroidism in the Southern Province

Congenital hypothyroidism (CH) is a condition that could be treated effectively if detected early. If not detected and treated early it causes server mental retardation. Treatment is simple, inexpensive and effective. With early detection and treatment, infants usually develop normally without mental handicaps and become productive members of society. It is identified as a very effective screening programme and the cost benefit of such a programme has shown to be very high.

The University of Ruhuna Nuclear Medicine Unit has kindly agreed to conduct analysis and reporting of a heal prick blood samples to screen for congenital hypothyroidism form all the Newborns in Southern Province.

The Family Health Bureau is collaborating with Provincial Health Authority, Teaching Hospitals and the Nuclear Medicine Unit, Faculty of Medicine, University of Ruhuna in implementing the Newborn Screening Programme in Congenital Hypothyroidism in the Southern Province.

Herewith I am attaching the Guidelines for Newborn Screening for Congenital Hypothyroidism. Please bring the contents of this circular and guidelines to the notice of all health workers in your Province/District/Institution and take necessary steps to implement this programme in Southern Province with immediate effect.

....
Dr Ajith Mendis
Director General Health Services

Dr. U. A. Mendis
Director General of Health Services
Ministry of Health
Ven. Baddegama Wimalawansa Thero Mawatha,
385, "Suwasiripaya"
Colombo 10.

Guidelines for newborn screening for congenital hypothyroidism

Congenital hypothyroidism (CH) is a treatable deficiency of the thyroid hormone that causes severe mental retardation and growth deficiency if it is not detected and treated early. Undetected at or near birth, CH clinically manifests itself too late for treatment to completely eliminate the mental and growth retardation that result. In many newborns CH may be diagnosed late or go completely undiagnosed resulting in unnecessary health, economic and social burdens for the family. Treatment is simple, inexpensive and effective. With early detection and treatment, infants usually develop normally without mental handicaps and become productive members of society. For optimum results treatment has to be initiated within two weeks.

Goal of the newborn screening programme; is to detect CH and begin treatment before the infant reaches the age of two weeks.

Newborn Screening system comprised of six essential components;

1. Education – Health professionals, parents and the general public
2. Screening – Proper timing, specimen collection, transport, laboratory testing and reporting
3. Early follow up – abnormal test notification, tracking and confirmatory testing
4. Diagnosis – Clinical and biochemical evaluation
5. Management – Counseling, treatment monitoring and long term follow-up
6. Evaluation – outcome monitoring and quality assurance throughout the system

Guidelines are given for each component, to be followed by the staff responsible for implementing the programme.

1. **Education** –

Professional education

From the outset, for the newborn screening for CH to be successful, health practitioners should be involved in its development. Health professionals should be educated on the benefits and operation of newborn screening as a preventive public health programme. They must also understand their role in this process.

Role of the Medical Officer of Health and the staff –

- Antenatal parent education on newborn screening for CH
- Postnatally deliver reports to parents, trace and refer newborns with abnormal reports to the Paediatricians for confirmatory diagnosis and treatment, follow up newborns with abnormal reports at home visits
- Educate mothers to inquire about report at second postnatal visit

Role of the hospital staff

- Counsel and educate parents
- Sample collection before the newborn is discharged
- Fill in the investigation request form correctly
- Store samples appropriately until dispatch to the laboratory
- Dispatch samples to the laboratory safely, on time
- Medical management of the detected case

Role of the laboratory staff

- Testing specimens
- Inform positive cases urgently to parents, Medical Officer of Health (MOH) office and hospital by telephone/fax/telegram and in addition to the letter
- Dispatch negative results to the relevant MOH office regularly
- Maintain laboratory standards and quality assurance systems

Parent education

In order to assist parents in understanding the importance and need for newborn screening, ongoing education is necessary. This has to be done at antenatal clinics in the MOH and at home visits by the Public Health Midwives (PHM). In the antenatal clinics this topic has to be addressed in the health education talk. The MOH has to update the staff on the newborn screening programme at the monthly conference.

*A parent education leaflet has to be prepared and given to the mother at the time of drawing blood.

2. **Screening**

Sample collection

Specimen has to be collected in the postnatal ward before the newborn is discharged from the hospital. It is strongly recommended that all newborns undergo screening by five days of age. The procedure should be carried out adhering to universal precautions.

All doctors and nurses in the postnatal ward and NICU/SCBU should be trained to do the heel prick by the Consultant Paediatrician in charge of the unit. The duty of performing the heel prick should be assigned to a nursing officer trained in heel prick in each unit in the morning shift at the same time when the BCG is given.

The heel prick – filter paper method is used for sample collection. Filter papers will be provided by the Laboratory.

Heel prick procedure –

Wash hands with soap and water before each collection.

Materials required – a pair of clean gloves, a sterile lancet with tip of a length less than 2.4 mm (approximately 2.0mm), sterile cotton balls, 70% isopropyl alcohol/surgical sprit and the filter paper card. Lancets should not be re used.

Preliminary preparation –

Complete the patient information form (supplied by the laboratory) using a ball-point pen. Avoid touching the filter paper collection area before, during and after the collection of blood spots. Adhere to universal precautions as in other procedures.

Location of the heel stick –

Use the lateral or medial plantar surface of the heel for the puncture site.

The heel puncture should only be taken from the shaded areas indicated by the arrows.

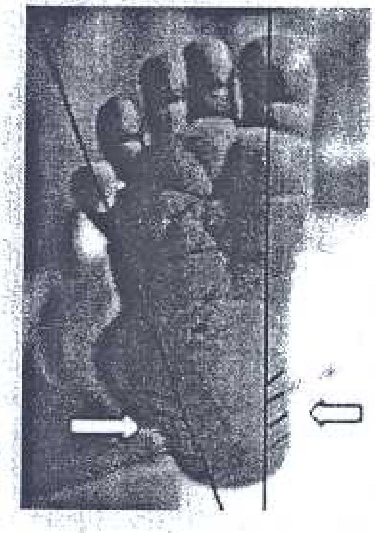

The heel puncture should only be taken from the shaded areas indicated by the arrows.

Heel preparation –
In order to increase blood flow, position the infant with feet at or below the level of the heart. Once ready clean the puncture site with a sterile cotton ball soaked in 70% isopropyl alcohol/surgical spirit and allow to air dry.

Puncture –Puncture the heel with a sterile lancet. Puncture device should not exceed 2.0 mm in length. For safety scalpel blades or needles should not be used as puncture device since depth of puncture or incision cannot be easily controlled and may lead to unwanted contact with the heel bone.
Steps should be taken to prevent pain during sample collection. Mother can start breastfeeding the baby which provide analgesia for the baby. Gentle stroking of the newborn and reassurance of the mother may be helpful.

Sample Collection –
After the heel has been punctured, wipe away the first drop of blood with a sterile gauze pad or cotton ball and allow a large drop of blood to form. Intermittently apply gentle pressure to the heel with the thumb and ease this pressure as drops of blood form. Avoid excessive milking or squeezing since this may cause specimen heamolysis or result in an admixture of tissue fluids with the specimen and might adversely affect test results. Allow a sufficient quantity of blood to soak through and completely fill one of the pre-printed circles on the filter paper. Apply blood to only one side of the filter paper. Examine both sides of the filter paper to ensure that the blood has uniformly penetrated and saturated the filter paper.

Post-collection
After collection elevate the baby's heel above the level of the heart while applying pressure to the wound with a sterile gauze pad or cotton swab until the bleeding stops. It is not advisable to apply adhesive bandages over the skin puncture sites of newborns.

Sample collection in special situations –
Samples of the babies who are admitted to the special care baby unit or the neonatal intensive care unit can be collected in the usual manner. If a newborn receives blood/blood products, the specimen should be collected before the transfusion. Screening can be performed on premature infants although caution must be taken when interpreting the results. It is recommended that preliminary screen be done by five days of age on all newborns even if the baby is in intensive care.

Specimen drying and transport -
Once the specimen is collected it should be air dried horizontally on a non absorbent open surface. It is important to keep the specimen horizontal to avoid migration of excess blood to a side of the circle. If the specimen is not transported to the laboratory on the same day keep it in the refrigerator in dry plastic/glass container with a lid in the lower compartment (8^0C) until it is transported. Moisture is detrimental to stability of dried blood spot specimen. Specimen packed in a dry envelop has to be transported to reach the laboratory within three days of collection. Nursing Sister/Nurse in-charge of the postnatal ward/NICU/SCBU is responsible for transporting the specimens to the laboratory on the stipulated date.

Record keeping

Request form
The request form supplied for this purpose has to completed and dispatched to the laboratory. Following is a specimen of the request form;

Newborn Screening for Congenital Hypothyroidism

(To avoid delays in processing complete this form accurately)

Hospital:______________________ Ward: BHT no: *

Date of Collection: ______________________ Time of Collection: ______________________

Mother's name: ______________________

Mothers Address: ____

Mothers contact telephone/mobile No; ______________________

Mothers resident MOH area : Mothers resident PHM area:

____

Babys Date of Birth: ______________ Time of birth: ______________

Sex of baby: ________ Birth weight: _____Kg POG: _______weeks

Signature of Meidcal officer

*In case of multiple pregnancies identify babies as T1, T2 etc.

Specimen Register

A specimen register has to be maintained in the postnatal ward NICU/SCBU in the following format. Information of all the specimens collected and dispatched has to be recorded in this register by the nursing officer at the time of collection.

Date of collection	Mother's BHT	Mother's Name	Mothers Home address	Mother's Telephone No	Mother's MOH area	Specimen sent by

Child Health Development Record

Make a note on the first page of the Child Health Development Record when the specimen is collected for newborn screening. Make sure that the Name of the hospital and BHT number is inserted here. In case of multiple pregnancies identify babies as T1, T2 etc.

TSH as the primary screen - The established Units (i.e., NMU Peradeniya & NMU Galle) had already developed their laboratory specific cut-off (whole blood TSH of 20.0mIU/mL) values for TSH assay with due consideration to the time after delivery and on the characteristics of the reagents in use and statistical testing of pilot projects. This will minimize false positive results which need unnecessary confirmatory testing. (box)

Abnormal results should be marked in red in the laboratory records.

Abnormal results should be informed by the analytical laboratory to the parents, MOH, hospital directly by telephone/fax to avoid any unforeseen delay. In positive cases the MOH should inform the PHM and trace the baby within 24 hours. Transmission of results of all specimens should be done directly to the MOH. All screening results should reach the postnatal clinics of the relevant MOH. PHMs should collect the reports of the newborns of their area and hand over to the parents during home visiting, after making a note in the CHDR (1st page or notes page) and the Birth and Immunization register.

3. **Diagnosis**

Once the screening tests have been completed and the results are known, it is important for babies who are presumptively at risk for a disorder to have confirmatory testing performed. This has to be done at a Paediatric clinic under the guidance of the Consultant Paediatrician according to the standard guidelines.

4. **Management**

Medical management should be closely monitored to ensure proper patient compliance and desired health outcome. Treatment of CH is lifelong and treatment compliance should be periodically monitored. Long term follow-up (reporting) helps ensure optimal health outcome and can be used to evaluate the screening programme.

1. Screening positive infants should have confirmatory measurements of FT4 and TSH in a serum sample as soon as possible.

2. All infants with low FT4 and elevated TSH have CH until proven otherwise.

3. Counseling of parents regarding the condition must be done by the Paediatrician.

4. Optimal cognitive outcome depends on both adequacy and timing of postnatal Thyroxine therapy.

5. Initial dose is 10-15mcg /kg /day Thyroxin should be given as a single daily dose in the early morning on an empty stomach for better absorption. Goal of therapy is to normalize T4 within 2 weeks and TSH within one month. The serum FT4 or total T4 should be in the upper half of the normal range during the first 3 years of life with a low normal serum TSH.

6. T4 value is used to adjust the dose, please note that the infants with low T4 (<10 mcg/dl) and high TSH (>15mcg/dl) during the first year have low IQ values.

7. All infants with confirmed CH should have an US Scan of the neck to visualize the thyroid gland.

8. Follow up is done with FT4 and TSH measurements. This should be done 4 weeks after the initiation of treatment and every 2 months for 6 months followed by every 4 months up to 3 years. Thereafter every 6 months until the growth is completed.

9. FT4 and TSH should be repeated after 4 weeks of adjusting the dose.

10. CH is permanent if the gland is absent or ectopic or if the serum TSH is above 10mIU/L after the first year of life.

11 If the clinician feels the diagnosis should be reviewed it should be done at 3 years of age.

Discontinue therapy for 30 days and retest with FT4/THS. Regarding individual cases with doubts the Paediatrician should seek expert opinion from an Endocrinologist.
